# Personalia

**Published:** 1914-04-04

**Authors:** 


### Personalia.

Mr. J. S. Horsley has been elected hon. secretary of the Brighton, Hove, and Preston Provident Dispensary, in place of the Rev. E. H. Enys, who has resigned.

Lieut.-Col. R. P. Simpson has resigned the hon. secretaryship of Petworth Cottage Hospital, which he has held for nineteen years. Dr. Eardley Wilmot has been elected to succeed him.

Dr. James Hill, M.B., Ch.B., B.A.O. (Belfast), has been promoted by the Camberwell Board of Guardians to the position of assistant medical officer at the Children's Homes and the Infirmary, which was vacant by the resignation of Dr. C. H. L. Rixon. Dr. Hill, who was house surgeon and ophthalmic officer at the Royal Infirmary, Sheffield, in 1913, previously held the post of assistant medical officer at Gordon Road Workhouse, Camberwell.

Mr. A. Drinkwater is resigning the post of honorary secretary to the Victoria Hospital, Kingston, as he is leaving the neighbourhood. He has held the post for four years.

Mr. Evan Spicer and Mr. Frederick Browning have joined the committee of the London Hospital.

Captain Harry Graham has been elected to the committee of management of the West End Hospital.

Two new members of the Public Pharmacists' and Dispensers' Association have been elected, namely, Mr. Geo. E. Town (Evelina Hospital) and Mr. T. H. Lloyd (Bethnal Green Infirmary).

Mr. W. Barlow, honorary secretary of the Aitken Memorial and Jubilee Cottage Hospital, Ramsbotham, has intimated his intention of resigning his post. Mr. Barlow has acted as honorary secretary for fourteen years.

Mr. O. E. Warburg has been elected chairman of the Public Health Committee of the London County Council, and Mr. L. Courtauld vice-chairman. The Public Health Committee is at present preparing a comprehensive scheme for the treatment of tuberculosis in London.

Mr. W. C. Johnson has been appointed an additional member of the Mental Hospitals and Mental Deficiency Committee of the London County Council.

